# Soil‐transmitted helminth infections associated with wastewater and sludge reuse: a review of current evidence

**DOI:** 10.1111/tmi.13076

**Published:** 2018-06-14

**Authors:** Isaac Dennis Amoah, Anthony Ayodeji Adegoke, Thor Axel Stenström

**Affiliations:** ^1^ Institute for Water and Wastewater Technology Durban University of Technology Durban South Africa

**Keywords:** soil‐transmitted helminths, wastewater reuse, sludge reuse, *Ascaris* spp, hookworm, *Toxocara* spp, helminthes transmis par le sol, réutilisation des eaux usées, réutilisation des boues, *Ascaris* spp, Ankylostome, *Toxocara* spp

## Abstract

**Objective:**

To review current evidence on infections related to the concentration of soil‐transmitted helminth (STH) eggs in wastewater, sludge and vegetables irrigated with wastewater or grown on sludge‐amended soils.

**Method:**

Search of Web of Science, Science Direct, PubMed and Google Scholar databases for publications reporting on STH egg concentration in wastewater, sludge and vegetables and for epidemiological studies on wastewater/sludge reuse and STH infections.

**Results:**

STH egg concentrations were variable but high in wastewater and sludge especially in developing countries. They ranged from 6 to 16 000 eggs/L in wastewater and from 0 to 23 000 eggs/g in sludge and far exceed limits set in the WHO guideline for wastewater/sludge reuse. Numbers of STH eggs on vegetables ranged from 0 to 100 eggs/g. The concentration of STH eggs in wastewater, sludge and vegetables therefore relates to risks of infection through different exposure routes.

**Conclusion:**

Epidemiological evidence reveals an increased prevalence of STH infections associated with direct exposure to wastewater or sludge (farmers) and consumption of vegetables grown on soil treated with it. This calls for increased efforts to reduce the adverse health impact of wastewater and sludge reuse in line with the WHO multi‐barrier approach.

## Abstract (fr)

**Objectif:**

Examiner les données actuelles sur les infections liées à la concentration d’œufs d'helminthes transmis par le sol (HTS) dans les eaux usées, les boues et les légumes irrigués avec des eaux usées ou cultivés sur des sols amendés par des boues.

**Méthode:**

Recherche dans les bases de données Web of Science, Science Direct, PubMed et Google Scholar pour des publications sur la concentration d’œufs d’HTS dans les eaux usées, les boues et les légumes et pour des études épidémiologiques sur la réutilisation des eaux usées/boues et les infections par les HTS.

**Résultats:**

Les concentrations d’œufs d’HTS étaient variables mais élevées dans les eaux usées et les boues, en particulier dans les pays en développement. Elles variaient de 6 à 16.000 œufs/L dans les eaux usées et de 0 à 23.000 œufs/g dans les boues et dépassent de loin les limites fixées dans les directives de l’OMS pour la réutilisation des eaux usées/boues. Le nombre d’œufs d’HTS sur les légumes variait de 0 à 100 œufs/g. La concentration d’œufs d’HTS dans les eaux usées, les boues et les légumes est donc liée aux risques d'infection par différentes voies d'exposition.

**Conclusion:**

Les données épidémiologiques révèlent une prévalence accrue des infections aux HTS associées à une exposition directe aux eaux usées ou aux boues (agriculteurs) et à la consommation de légumes cultivés sur des sols traités à la boue. Cela appel à accroitre les efforts pour réduire l'impact négatif de la réutilisation des eaux usées et des boues sur la santé, conformément à l'approche multi‐barrières de l’OMS.

## Introduction

Wastewater and sludge reuse in agriculture have been promoted as a concept of sustainable development 1. Wastewater may be used for irrigation either directly, or indirectly through the use of wastewater‐contaminated surface water. Contamination of surface water with wastewater may occur due to poor infrastructure, such as non‐treated effluents, leaky sewage pipes and faulty wastewater treatment plants 2. The increase in the use of wastewater and sludge globally is driven by rapid urbanisation, growing water shortage and the benefits associated with the practice as irrigation water 1, 2, 3, 4. With approximately 330 km^3^ of municipal wastewater produced per year 5, it is a reliable alternative water source for irrigation. Wastewater and sludge reuse is most predominant in developing and arid countries, but has also been documented in Northern America, Europe and Australia 2, 6, 7, 8. Globally, wastewater‐irrigated farms are estimated to cover 5–20 million hectares, with higher proportions in China 9 and Mexico 10. When also accounting for wastewater‐contaminated surface water, the total land area irrigated with wastewater is substantially higher 11. The use of sludge is encouraged due to its high nutrient and organic material contents 12. It is mostly applied as a soil amendment 13 to improve water retention of soil 14, 15. Sludge may also be used to rehabilitate degraded, exhausted and burnt soil 16, 17.

Despite the many benefits of wastewater and sludge use in agriculture, these practices can have adverse impacts on human health 18, 19. Wastewater may contain contaminants that are harmful to health, such as metalloids/metals 20, 21, excess nutrients, hormones 22, 23, 24, organic compounds such as pesticides, components of consumer products, pharmaceuticals and personal care products 25, 26, 27 and, most importantly, pathogenic microorganisms 28, 29, 30, 31, 32.

Among the microbial pathogens, soil‐transmitted helminths (STHs) are of the most important health concern in wastewater and sludge reuse 19 especially in endemic regions, mainly due to their persistence in the environment and the low infectious dose 33, 34, 35, 36, 37. Concentration of STH eggs in wastewater/sludge is an indication of the health hazard of its application 38, 39. Despite the adoption of the WHO wastewater/sludge reuse guidelines and the development of local guidelines, where the suitability of these (wastewater and sludge) for reuse is covered, increased STH infections for different populations due to wastewater/sludge reuse continue to be reported. This review therefore presents the current evidence in relation to concentration of STH eggs in wastewater and sludge and reported adverse health effects (STH infections).

## Methods

### Search strategy

This review was based on a literature search of Web of Science, Science Direct, PubMed and Google Scholar databases. The keywords and word strings used were soil‐transmitted helminths OR intestinal parasites OR *Ascaris* spp OR hookworm OR *Trichuris* spp OR *Toxocara* spp OR *Taenia* spp AND wastewater reuse OR sludge reuse. The organism search strings were repeated with AND compost OR vegetables OR crops OR plants; as well as with AND soil OR urine diversion (UD) toilet waste OR biosolids. Although *Taenia* spp is not a STH it was added to the review due to the similarity in survival characteristics in the environment, its epidemiology and transmission route with STHs 40.

Original articles reporting on STH egg concentrations in wastewater, sludge, compost and crops or vegetables were considered. Only peer‐reviewed papers published in English were included. There were no restrictions on publication year or geographic location.

Article titles and abstracts were assessed to determine their suitability for inclusion in this review. Studies included can be categorised into publications on the concentration of STH eggs in wastewater and sludge reused in agriculture; publications that reported the concentration of these eggs on vegetables grown with wastewater or sludge; and publications that directly measured the impact of wastewater/sludge reuse on STH infections using epidemiological methods. Studies that reported infections other than STHs and *Taenia* spp were not included.

### Data extraction and analysis

The following information was collected from each article considered for this review: (1) geographic location of the study; (2) concentration of STH eggs in samples studied; (3) type of STH reported; (4) target population studied; (5) infection levels reported in each exposed population.

STH egg concentrations in wastewater, sludge and on vegetables from articles and reports were collated and captured in tables for easy representation. Measured impacts of wastewater/sludge reuse on STH infections were also collated and the reported odds of infection, odds ratios or prevalence of infection from exposure captured in tables.

## Results

**Figure 1:**
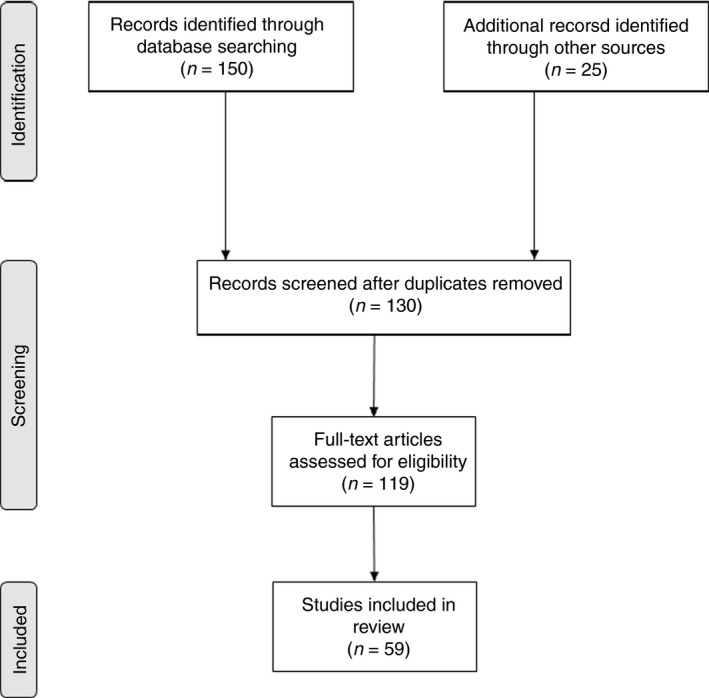
Number of articles obtained through the search process and the studies reviewed based on eligibility criteria.

The search yielded 175 articles, 150 from the search strings used and 25 from manual search. Of this number, 59 were included in this review. Figure 1 shows the process followed in arriving at the final number of articles reviewed. Of the 59 articles reviewed, 25 reported on the concentration of STH eggs in wastewater and sludge, 19 on STH contamination of vegetables irrigated with wastewater or grown on sludge‐amended soils and 15 reported on the direct epidemiological link between wastewater/sludge reuse and STH infections.

### Concentration of STH eggs in wastewater and sludge

STH eggs are a major group of pathogens of concern with concentrations in wastewater and sludge that vary between locations due to differences in infection prevalence or intensity in the connected population. An estimated 3000 eggs/L may be found in wastewater from endemic regions 41, 42. Table 1 summarises reported concentrations of STH eggs found in wastewater and sludge. STH egg concentrations in developing countries are generally higher than in developed countries.

**Table 1:** Concentration of STH eggs in wastewater and sludge from different locations

Country	Wastewater (eggs/L)	Sludge (eggs/g)	References
Egypt	6–42	Mean: 67; Maximum: 735	84
Ghana	12.9–15.1	13–94	55, 85, 86
Morocco	840	3.3–13.3	87, 88
South Africa	772	25–185	89, 90, 91
Tunisia	15–30	0–4	87, 92, 93, 94
Brazil	166–202	75	85, 95
United States	1–16	2–776	85, 96, 97, 98
Mexico	6–98	73–177	85, 99
Peru	115–273	60–260	87, 100
Japan	80	1–51	87
China	840	2300	87
Syria	800		87
Vietnam	450–16000		101
Pakistan	142–558		102
Ukraine	60	No data	87
France	9	5–7	85, 103
Germany	No data	<1	85
Great Britain	No data	<6	85
Spain	0–1	867	104, 105, 106, 107

### Concentration of STH eggs on vegetables irrigated with wastewater or grown on sludge‐amended soil

Contamination of vegetables with STH eggs due to wastewater and sludge reuse occurs when these are used for irrigation or fertilisation. Local factors such as impact of UV light, rain pattern and withholding time between irrigation/fertilisation and harvest determine the concentrations and risk. The impact of these factors on STH egg levels on vegetables has rarely been addressed 43, 44, 45. Concentrations of STH eggs ranged from 0.027 eggs/100 g 43 to 10 eggs/100 g 44 (Table 2). It is noteworthy that some of these did not report concentration of STH eggs but rather the prevalence of contamination in a particular region. Also, contamination may not always be from wastewater or sludge reuse but in some cases may have been from post‐harvest handling of the produce.

**Table 2:** Prevalence and concentration of STH and other helminth eggs contamination on vegetables

Type Of STH	Concentration	Location	References
*Ascaris* spp.	0.018 eggs/100 g	Morocco	43
	0.027 eggs/100 g		
	0.27 eggs/100 g (Coriander)		
	0.46 eggs/100 g (Mint)		
	0.07 eggs/100 g (Carrots)		
	0.16 eggs/100 g (Radish)		
*Ascaris* spp.	1–3 eggs/100 g (Lettuce)	Turkey	108
	0–3 eggs/100 g (Parsley)		
	0–2 eggs/100 g (Spinach)		
All helminth eggs	10 eggs/100 g (water spinach)	Cambodia	44
All helminth eggs	2.3 eggs/100 g (Lettuce)	Ghana	109
*Ascaris lumbricoides,* Hookworm*, Enterobius vermicularis, Trichuris trichiura, Taenia* and *Strongyloides* larvae	0.8–3.7 eggs/100 g (lettuce)	Ethiopia	110
*Taenia* spp, *Ascaris* spp*, Toxocara* spp and *Strongyloides* eggs.	8.4 eggs/100 g (mint, coriander, alfalfa).	Morocco	45
All STHs	32.6% (of 304) (a variety of vegetables)	Iran	111
*Ascaris* spp	2% (of 141)^a^	Iran	112
*Ascaris* spp*, Trichuris trichiura* and Hookworm	44.2% (of 172) (a variety of vegetables)	India	113
All STHs	57.8% (of 199) (a variety of vegetables)	Nigeria	114
*Ascaris* spp	19–96% (of 126)	Libya	115
*Toxocara* spp	3–48% (of 126)		
*Taenia* spp	6–30% (of 126) (A variety of vegetables)		
*Ascaris* spp., Hookworm, *Trichuris* spp., *Taenia/Echinococcus* spp., and *Strongyloides stercoralis*	8.2% (of 1130 for lettuce)	Nigeria	63
	2.0% (of 1130 for cabbage)		
	1.0% (of 1130 for eggplant)		
	1.3% (of 1130 for carrot)		
	2.3% (of 1130 for cucumber)		
All helminths	6.3% (of 111) (A variety of vegetables)	Turkey	64
All helminths	36.9% (of 118) (A variety of vegetables)	Palestine	116
Intestinal parasites	61% (of 168) (lettuce)	Ghana	117
	18% (of 168) (tomato)		
Intestinal parasites	13.5% (of 260)^a^	Sudan	118
All STHs	16.2% (of 270)^a^	Saudi Arabia	65
Intestinal parasites	8.4% (of 383)^a^	Iran	119
Intestinal parasites	14.6% (of 383)^a^	Iran	120

a Not specified.

### Evidence of STH infections associated with wastewater and sludge reuse

Direct exposure to wastewater or sludge during their application may result in STH infections, and consumption of the farm produce may result in infections due to the contamination. Several epidemiological studies established the association between wastewater/sludge reuse and STH infections (Table 3).

**Table 3:** Studies reporting on the association between wastewater/sludge use in agriculture and STH infections

Author/Year	Target group	Practice	Health risk and conclusions
Pham‐Duc *et al*. 121	Farming households	Wastewater and excreta	Contact with wastewater was a significant risk factor for helminth infection (OR = 1.5, 95% CI 1.1–2.2) in general and also specifically for *Ascaris lumbricoides* infection (OR = 2.1, 95% CI 1.4–3.2). Significant risk factors for *Trichuris trichiura* infection include the use of human excreta (OR = 1.5, 95% CI 1.0–2.3)
Yajima *et al*. 57	Community members	Human excreta only	Consumption of vegetables fertilised with human excreta resulted in high helminth infection rate
Trang *et al*. 74	Farming households	Wastewater	No significant association was found between wastewater exposure and helminth infections
Trang *et al*. 122	Adults and children	Wastewater and human excreta	Wastewater exposure did not pose a significant risk for helminth infection
Nguyen *et al*. 73	Women	Excreta	The use of untreated faeces as fertiliser was significantly associated with infection with *A. lumbricoides* (OR = 1.2, 95% CI 1.0–1.6)
Van der‐Hoek *et al*. 1	Community members	Human excreta only	The use of human excreta as fertiliser was a significant risk factor for hookworm infection, especially among adult women
Gumbo *et al*. 123	Male farmers	Wastewater	Farmers using wastewater for irrigation had a prevalence ratio of 1.50 for hookworm infections
Habbari *et al*. 61	Children	Wastewater	Significant increase in prevalence of ascariasis for exposed children
Bouhoum and Schwartzbrod 124	Children	Wastewater	Higher prevalence among exposed of any helminth infection (73% exposed *vs*. 30% unexposed), *Ascaris* infection (33% *vs*. 2%), and *Trichuris* infection (17% *vs*. 2%)
Blumenthal *et al*. 61	Agricultural workers and their family members	Wastewater	Higher prevalence of *Ascaris* infection among exposed compared to unexposed for children. Children under 5 years had higher odds of infection (OR = 18.0) than children above 5 years (13.5)
Cifuentes *et al*. 125	Agricultural workers and their family members	Wastewater	Higher prevalence of diarrheal disease (30% *vs*. 23%) and *Ascaris* infection (15% *vs*. 3%) for exposed children
Amoah *et al*. 54	Agricultural workers and their family members	Wastewater	Increased odds of infection for farmers for both *Ascaris* spp and hookworm for farmers and family members as compared to unexposed populations, especially in the raining season
Pham‐Duc *et al*. 18	Farming households	Wastewater	People having close contact with wastewater polluted surface water had a higher risk of helminth infections compared with those without contact
Fuhrimann *et al*. 72	Community members	Wastewater	High prevalence of intestinal parasite infections for peri‐urban farmers, using wastewater for irrigation, as compared to other groups

## Discussion

STH eggs in wastewater and sludge pose a threat to human health upon direct or indirect exposure. Concentration of these eggs in faecal materials varies greatly depending on the infection levels within the connected populations 46, 47. Infected individuals can excrete 10^2^–10^4^ eggs/g daily, contributing to high concentrations in wastewater and sludge 48. Human faecal matter is the main concern, but impact from animals such as dogs and cats is also important where their faeces may contain eggs of *Toxocara* spp, which could lead to zoonotic infection. These eggs (*Toxocara* spp) are predominantly found in investigations of soil and playgrounds in developed countries, especially where pets are allowed 49, 50, while their abundance may be more generally occurring in many developing countries. The generally high concentration of STH eggs in wastewater and sludge is reflected by the infection prevalence and is of concern especially in reuse scenarios. Reuse of the wastewater and sludge is one major route through which exposure to the eggs may occur and affect exposed farmers directly, while indirectly impacting broader groups such as community members and consumers of crops. Infection risks are based on a single STH egg dose. The concentrations of STH eggs reported in wastewater and sludge as presented in Table 1 reflect the risks based on their further deposit or reuse linked to human direct or indirect exposure. Exposure is mainly through the oral route for most STHs and additionally through skin penetration for hookworms. The concentrations found in the majority of the reported investigations (Table 1) exceed the WHO guideline values for wastewater/sludge reuse and result in infections far above the health target of ≤10^−6^ disability adjusted life years (DALYs) 19. Direct exposure to wastewater or sludge during application is the main route of exposure for farmers. The informal nature of wastewater or sludge reuse in many countries makes regulation difficult and also hinders implementation of effective public health awareness campaigns to reduce the impact of the practice on the health of farmers and the general population.

Exposure to soil containing STH eggs may also result in increased infections. Due to the importance of soil in the transmission of these infections, several studies have focused on the prevalence and concentration of STH eggs in public parks, beaches, backyards and farmlands 51. The prevalence and concentration of these eggs in soil varies greatly from 1.1 eggs/g 52 to 454.5 eggs/g 53. Accumulation of STH eggs in farm soil occurs due to multiple applications of wastewater and sludge to the same parcel of land 51, 54. As this accumulation persists over time, the risk of infection is not diminished due to the latency period. Coupled with the persistence of these eggs in the environment of 10–12 months 55, STH egg concentrations on farm soil could be higher than concentrations in the wastewater or sludge resulting in higher infection risks 54, 56. For instance, Amoah *et al*. 54 reported an average concentration of 3.70 (±0.23) eggs/g for *Ascaris* spp and 2.01 (±0.23) eggs/g for hookworm on farms in Ghana. Fixed time periods for survival may be difficult to give as conditions may vary greatly. Due to the low infection dose (essentially a male and female worm for multiplication in the body), a conservative approach has been taken with an essentially low occurrence of eggs in environmental samples.

Direct exposure to wastewater and sludge has been reported as the main route of infection with STHs associated with reuse. However, Yajima *et al*. 57 reported an increased risk of STHs infections from consumption of vegetables fertilised with sludge rather than through direct exposure to sludge. The contamination of vegetables with STH eggs due to wastewater reuse is mainly dependent on the quality of the wastewater, the irrigation method used and the type of crops. WHO wastewater reuse guidelines relate the risk to the likelihood of contact, where for example the use of drip irrigation for high crops is given a reduction in the likelihood of infection risks of 4 log units of pathogens. A comparative value of 2 log units for low crops applies. A higher reduction in pathogens could be achieved with the use of subsurface irrigation, with 6 log units 19. If instead sprinkler irrigation is used, this results in a higher risk due to the contact between potential STH eggs in water droplets and the crops, causing higher contamination of the vegetables. High ejection sprinkler irrigation may also result in exposure risks for communities residing close to the irrigation sites due to aerosols as reported in several earlier studies 58, 59, 60. Furrow or flood irrigation may also increase the possibility of direct contact with the wastewater by farmers, increasing risks of infections 61, 62.

Contamination of vegetables with STH eggs from wastewater‐irrigated fields has been reported extensively 63, 64, 65, 66, 67, 68 (Table 2). The relative impact of contamination from other activities or routes parallel to wastewater/sludge reuse has not been demonstrated clearly. Contamination levels of STH eggs on vegetables are largely dependent on the quality of the wastewater/sludge as well as the irrigation method. Post‐harvest practices such as hygiene of market women and even cleanliness of the markets during retail and transportation may result in additional contamination of the vegetables with STH eggs 69. Uga *et al*. 70, reported that a high contamination of vegetables from markets occurred with both animal and human parasites. This could be attributed to both contaminations during cultivation and handling and storage practices at the various markets. Shuval *et al*. 71 reported that eggs of common STHs could survive on crops 4–12 months. These findings were instrumental in the development of the first WHO wastewater reuse guidelines.

Despite the established estimated risk levels of STH infections from wastewater/sludge reuse through QMRA, only a small number of studies have applied epidemiological assessments and methods to accurately measure the contribution of wastewater/sludge reuse to STH infections (Table 3).

Independently of that, a review of the scarce literature shows an overwhelming evidence of increased risks of STH infections due to wastewater/sludge reuse 1, 18, 54, 72, 73 even if some studies were unable to establish links. For instance, Trang *et al*. 74 found no increase in STH infections from exposure to wastewater in Vietnam. The interlinkages between different contamination sources and transmission routes are essential in these types of studies. Pham‐Duc *et al*. 75 found that hygiene was the main pre‐disposing factor to an increased risk of parasitic infections (mainly protozoan) rather than exposure to wastewater or sludge. The epidemiological studies highlight a high incidence of STH infections among farmers and consumers of vegetables irrigated with wastewater or on sludge amended soil. The epidemiological approach may be more accurate in determination of risks of STH infections associated with wastewater/sludge reuse than the QMRA, but is dependent on large enough sample sizes, a well‐documented background level of disease and appropriate control groups. In these situations, the QMRA approach is simpler and can be interlinked with the epidemiological studies. QMRA therefore is a valuable predictive tool and has been used extensively in estimating the risks of STH infections from wastewater or sludge reuse 31, 56, 76, 77, 78, 79, 80, 81. The estimated risks are affected by several assumptions, which do not affect the epidemiological assessments to the same extent, like the dose of STH eggs ingested by exposed individuals (either assumed or calculated) and to a large extent the dose–response model used. These variations could result in under or over estimation of the associated risks. The use of different dose–response models to estimate infection risks from ingestion of the same dose would result in different risks estimates which creates a challenge for comparison. Despite the usefulness of the epidemiological methods as a golden standard, limitations also relate to the costs involved which is far higher than predictive estimates. These costs for epidemiological studies might be the driving force behind the increase use of the QMRA method in estimation of risks.

The use of either QMRA or epidemiological methods has established that wastewater or sludge reuse contributes significantly to increased STH infections. These risks are mainly due to the high concentration of STH eggs in the wastewater or sludge, which calls for the adoption of effective strategies for risks reduction and/or management. The WHO wastewater/sludge reuse guidelines suggested protective measures that will ensure a 6–7 log unit reduction in pathogen concentrations, thereby reducing risks of infections for both farmers and consumers. These measures, referred to as t*he multiple barrier approach*, include the following:

- Wastewater/sludge treatment: The concentration of STH eggs after treatment varies depending on the treatment technology. For instance, the highest reduction in helminth eggs (1–3 log units) has been reported with low rate biological processes such as waste stabilisation ponds, wastewater storage and treatment reservoirs and constructed wetlands 19. Therefore, reuse scenarios could be developed based on the type of treatment technology available or the treatment technology could be chosen based on the intended reuse. Background values like the ones summarised in Table 1 can then be applied in comparative assessments of the reduction efficiency and variability.

- Control of human exposure to the pathogens in the wastewater/sludge: This could be achieved through the use of protective gear such as boots, gloves, nose masks, etc. However, this is rare, especially in developing countries, where the practice is common, due to the financial constraints. In this context, both background information of contamination levels (Table 1) and follow‐up epidemiological evidences (Table 3) are of importance and should be linked with risk‐reduction strategies.
- Crop restriction: This measure helps ensure that the choice of crops irrigated or grown is dependent on the quality of wastewater/sludge so as to protect health. However, the WHO reuse guidelines acknowledge that this will not be possible in countries where a monitoring regime is not in place. Wastewater/sludge reuse in most countries is informal and driven by economic reasons; therefore, this barrier may not be effectively implemented. By a combination of information on incidence of STH infections in an area or region, risk assumptions may be reached, which combined with irrigation practices and potential occurrence (Tables 1 and 2) can give an indication for handling practices.
- Wastewater application technique: The type of irrigation technique plays a major role in contamination of crops with pathogens from wastewater. Different irrigation methods carry varying risks in relation to contamination of vegetables, exposure of farmers and communities living close to irrigation sites.
- Cessation of irrigation before harvest: Pathogens will naturally die depending on their survival ability; therefore, cessation of irrigation days before harvest has been suggested to result in reduction of contamination. However, this might not necessarily result in reduction of viable STH eggs due to their high persistence in the environment.
- Food preparation techniques: The pathogen reduction possible depends on the food preparation method, for instance washing in water and hypochlorite solution may result in 1–3 log units reduction, peeling of fruits and root vegetables 2 log reduction and cooking may result in 5–6 log unit reduction in pathogens. The food preparation method may therefore be instrumental in public health protection 19 where a higher and comparative assessment as related to the epidemiological outcomes (Table 3) would give a better decision base for the future.

The WHO wastewater/sludge reuse guideline also recommends a guideline value of ≤1 helminth egg per gram or litre of sludge or wastewater intended for unrestricted agriculture. Some researchers suggest the reduction of this guideline value to ≤0.1 helminth egg per gram or litre, especially in situations where children are exposed 82. However, in resource‐limited countries where prevalences of STH infections are high and causing high concentrations of eggs in the wastewater/sludge, this may not be achievable. In such situations, the use of the QMRA approach may provide an option to determine the least quality required for specific uses. This may result in the preparation of different application guidelines depending on the quality achievable with the treatment options available in such settings. The multiple barrier approach should always be advocated for in these situations.

The Sanitation Safety Plan (SSP) Manual prepared by WHO 83 makes it easier to implement these wastewater/sludge reuse guidelines by presenting a stepwise approach to incorporation of reuse scenarios to sanitation. This manual presents six modules for the planning of sanitation systems, from generation through to collection, transportation, to treatment and reuse. The SSP manual accounts for risks of reuse through the use of the QMRA approach which applies the principle of hazard analysis and critical control points (HACCP) based on the Stockholm framework for preventive risk assessment and management. The WHO wastewater/sludge reuse guidelines as well as the SSPs provide means for effective reuse of wastewater and sludge whiles protecting public health, but they must be adapted to the local context so as to ensure usefulness. In this context, better background information on occurrence, like presented in Tables 1 and 2, and a broader epidemiological evidence base (Table 3) can be useful in decision‐making and management of reuse schemes.

## Conclusion

STH eggs in wastewater and sludge continue to pose serious risks for farmers and other farm workers, including consumers through contamination of vegetables with these eggs. From the scarce studies on epidemiological links between wastewater/sludge reuse and STH infections, it can be concluded that this practice contributes significantly to increased infections. Implementation of the WHO wastewater/sludge reuse guidelines and sanitation safety plans may be instrumental in reducing the risks associated with wastewater/sludge reuse. These guidelines present interventions that may provide additional layers of protection even in cases where the quality of wastewater or sludge being used is poor. The SSPs also provide steps that could be adapted to local contexts for safe reuse of wastewater and sludge. We suggest the adaptation of the WHO wastewater/sludge reuse guidelines and SSPs to local contexts by involving farmers, communities and policy makers to ensure their effective implementation.
